# Covariance of Charged Amino Acids at Positions 322 and 440 of HIV-1 Env Contributes to Coreceptor Specificity of Subtype B Viruses, and Can Be Used to Improve the Performance of V3 Sequence-Based Coreceptor Usage Prediction Algorithms

**DOI:** 10.1371/journal.pone.0109771

**Published:** 2014-10-14

**Authors:** Kieran Cashin, Jasminka Sterjovski, Katherine L. Harvey, Paul A. Ramsland, Melissa J. Churchill, Paul R. Gorry

**Affiliations:** 1 Center for Biomedical Research, Burnet Institute, Melbourne, Australia; 2 Department of Microbiology and Immunology, University of Melbourne, Melbourne, Australia; 3 Department of Microbiology, Monash University, Melbourne, Australia; 4 Department of Surgery (Austin Health), University of Melbourne, Melbourne, Australia; 5 Department of Immunology, Monash University, Melbourne, Australia; 6 School of Biomedical Sciences, CHIRI Biosciences, Faculty of Health Sciences, Curtin University, Perth, Australia; 7 Department of Medicine, Monash University, Melbourne, Australia; 8 Department of Infectious Diseases, Monash University, Melbourne, Australia; University of Cape Town, South Africa

## Abstract

The ability to determine coreceptor usage of patient-derived human immunodeficiency virus type 1 (HIV-1) strains is clinically important, particularly for the administration of the CCR5 antagonist maraviroc. The envelope glycoprotein (Env) determinants of coreceptor specificity lie primarily within the gp120 V3 loop region, although other Env determinants have been shown to influence gp120-coreceptor interactions. Here, we determined whether conserved amino acid alterations outside the V3 loop that contribute to coreceptor usage exist, and whether these alterations improve the performance of V3 sequence-based coreceptor usage prediction algorithms. We demonstrate a significant covariant association between charged amino acids at position 322 in V3 and position 440 in the C4 Env region that contributes to the specificity of HIV-1 subtype B strains for CCR5 or CXCR4. Specifically, positively charged Lys/Arg at position 322 and negatively charged Asp/Glu at position 440 occurred more frequently in CXCR4-using viruses, whereas negatively charged Asp/Glu at position 322 and positively charged Arg at position 440 occurred more frequently in R5 strains. In the context of CD4-bound gp120, structural models suggest that covariation of amino acids at Env positions 322 and 440 has the potential to alter electrostatic interactions that are formed between gp120 and charged amino acids in the CCR5 N-terminus. We further demonstrate that inclusion of a “440 rule” can improve the sensitivity of several V3 sequence-based genotypic algorithms for predicting coreceptor usage of subtype B HIV-1 strains, without compromising specificity, and significantly improves the AUROC of the geno2pheno algorithm when set to its recommended false positive rate of 5.75%. Together, our results provide further mechanistic insights into the intra-molecular interactions within Env that contribute to coreceptor specificity of subtype B HIV-1 strains, and demonstrate that incorporation of Env determinants outside V3 can improve the reliability of coreceptor usage prediction algorithms.

## Introduction

Human immunodeficiency virus type 1 (HIV-1) enters cells after binding of the viral envelope complex (Env) to CD4 and one of two chemokine coreceptors, CCR5 or CXCR4, on the host cell [1]–[5]. The transmission and establishment of HIV-1 infection is associated with virus populations that utilize CCR5 (R5 viruses), and the majority of patients maintain R5 viruses throughout infection [6], [7]. However, in approximately 40% of subtype B HIV-1 (B-HIV) infections, viruses that utilize CXCR4 (R5X4 and X4 viruses) emerge, which is associated with rapid CD4+ T-cell decline and onset of acquired immunodeficiency syndrome (AIDS) [8]–[13].

Maraviroc (MVC) is a CCR5 antagonist that is used in combined antiretroviral therapy (cART) regimens to inhibit HIV-1 entry by binding to- and altering the conformation of CCR5 such that it is no longer able to interact with CD4-bound gp120 [14]-[16]. MVC can only be administered to HIV-1 patients who exclusively harbour R5 virus populations in their plasma [14], [17], [18]. Consequently, the ability to determine coreceptor usage of patient-derived HIV-1 isolates is clinically important. The gold standard approaches for establishing coreceptor usage of HIV-1 are phenotypic cell entry assays, whereby infectious viruses pseudotyped with patient derived Envs are used to infect cell lines expressing CD4 together with CCR5 or CXCR4 [16], [19]. However, the cost, turn-around time and specialized nature of these assays have limited access to MVC for many patients worldwide. Consequently, *in silico* coreceptor usage prediction algorithms have emerged as an inexpensive, rapid and relatively straightforward means of determining HIV-1 coreceptor usage.

The HIV-1 Env complex is composed of trimers of non-covalently associated gp120 and gp41 glycoproteins. Gp120 consists of five hyper-variable loop regions (V1–V5) interspersed by five relatively conserved constant regions (C1–C5) [20]–[22]. Extensive mutagenesis studies have shown that the major determinants of HIV-1 coreceptor usage lie within the gp120 V3 loop, and characteristic sequence alterations within this region are the basis for currently available genotypic algorithms that predict HIV-1 coreceptor usage [2], [22]–[26]. For example, the “11/25 rule” predicts CXCR4-usage based on the presence of positively charged amino acids at positions 11 and/or 25 in V3 (Env positions 306 and 322, respectively) [23], [27]. More sophisticated *in silico* V3 sequence-based genotypic algorithms such as geno2pheno_[coreceptor]_ (G2P) [28] and WebPSSM [25], [29] can predict coreceptor usage of patient isolates and MVC treatment outcomes with reasonable accuracy [30]–[33]. However, V3 sequence-based algorithms are inherently limited in their predictive capabilities given that determinants of coreceptor usage may also lie within the V1/V2 and C4 Env regions [26], [34]–[39].

As the cost, breadth, and yield of HIV-1 Env sequencing techniques rapidly improves, incorporation of Env regions outside V3 to enhance the predictive accuracy of genotypic algorithms is becoming increasingly viable. Unfortunately, few studies have investigated regions outside V3 that differentiate CXCR4-using from R5 HIV-1 strains, largely due to the relative paucity of phenotypically characterized full-length Env sequences available for analysis. Here, we have performed the most extensive analysis of phenotypically characterized HIV-1 Env sequences to date, in order to elucidate determinants of coreceptor usage outside V3 that contribute to coreceptor specificity.

## Materials and Methods

### HIV-1 sequence data sets

For this study we utilized HIV-1 sequences from the Los Alamos HIV Database (http://www.hiv.lanl.gov/) and from a recently characterized HIV-1 subtype C (C-HIV) cohort [26], randomly selecting one sequence per patient using a random number generator to avoid resampling bias (as of April 2014; n = 11,315: 361 subtype A, 941 subtype A1, 3907 subtype B, 3489 subtype C, 474 subtype D, 211 subtype G, 1154 CRF01_AE, 410 CRF02_AG, 267 CRF07_BC and 101 CRF08_BC). Sequences were derived from patients residing in diverse geographical locations such as Africa, India, South America, USA, Europe, Southeast Asia, Central Asia and Australia. For analysis of phenotypically characterized sequences, we collected all sequences that were previously determined to use CCR5 and/or CXCR4 through the use of phenotypic cell entry assays, and randomly selected one sequence per patient per phenotype. Phenotypically characterized sequences were grouped as either R5 or “CXCR4-using” (R5X4 and X4 sequences). Altogether we collected 43 CXCR4-using (23 R5X4, 20 X4) and 223 R5 subtype B (B-HIV) Env sequences, an additional 32 CXCR4-using (23 R5X4, 9 X4) and 70 R5 B-HIV sequences containing at least V3 and position 440, 34 CXCR4-using (23 R5X4, 11 X4) and 193 R5 C-HIV sequences containing at least V3 and position 440, 27 CXCR4-using (18 R5X4, 9 X4) and 53 R5 subtype D HIV-1 (D-HIV) sequences containing at least V3 and position 440, and 32 CXCR4-using (13 R5X4, 19 X4) and 107 R5 CRF01_AE HIV-1 sequences (AE-HIV) containing at least V3 and position 440.

### Sequence analysis

Sequences were aligned using CLC Main Workbench version 6.5. VESPA analysis was performed using the “Viral Epidemiology Signature Pattern Analysis (VESPA)” tool available on the Los Alamos HIV Database. VEPSA identifies sites where the consensus sequence differs between two groups and calculates the frequency of each amino acid at these sites. The default VESPA threshold of zero was used, which reports the majority amino acid at each site, in order to identify sites where the most frequent amino acid differs between R5 and CXCR4-using sequences.

### Statistical analysis

P-values for VESPA analysis were calculated using a Fisher's exact t-test (two-tailed). Correlation tables were analyzed for statistical significance using the chi-square test (χ^2^). Statistical tests and area under the receiver operating characteristic curve (AUROC) calculations were performed according to [40] using Prism version 6 (GraphPad Software Inc., San Diego, CA). AUROC calculations were performed to quantify the sensitivity/specificity profiles of genotypic algorithms. Statistical analysis (two-tailed t-test, p≤0.05 considered significant), comparing the difference between two AUROC's, was performed to determine whether or not inclusion of the “440 rule” significantly improved the AUROC of a genotypic algorithm, relative to the AUROC of the unmodified version.

### Coreceptor usage prediction algorithms

The “11/25 rule” predicts CXCR4-usage based on the presence of Arg or Lys at V3 positions 11 and/or 25 (Env positions 306 and 322 respectively) [23], [27]. The support vector machine based algorithm geno2pheno_[coreceptor]_ (G2P) [28] is available at http://coreceptor.bioinf.mpi-inf.mpg.de/. The position-specific scoring matrix based WebPSSM algorithms [25], [29] are available at http://indra.mullins.microbiol.washington.edu/webpssm/. The CoRSeq_V3-C_ algorithm [41], which predicts coreceptor usage based on the presence of V3 amino acid alterations that are characteristic of CXCR4-using and R5 subtype C HIV-1, is available at http://www.burnet.edu.au/coreceptor/. The “440 rule” involves rescreening all sequences predicted to be R5 by the unmodified genotypic algorithm for the presence of Asp or Glu at position 440, the presence of which results in a “CXCR4-using” prediction.

### Env structural modeling

Env structural models were developed using the crystal structure of CD4-bound YU2 gp120 containing V3 that has been docked with the nuclear magnetic resonance (NMR) structure of an N-terminus peptide of CCR5 (^7^SPIYDINYY^15^; sulfated tyrosines at positions 10 and 14 amino acids are underlined), which was kindly provided by P. D. Kwong [20]. We purposely used this structure of gp120 because it permitted the analysis of potential interactions between residues at gp120 positions 322 and 440 and the CCR5 N-terminus, and hence how they may contribute to coreceptor specificity. Mutations at positions 322 in V3 and 440 in C4 were introduced using the Mutate Protein protocol that we have described previously [42]. Briefly, point mutations were introduced via sequence alignment, and harmonic constraints were applied prior to optimization using the Steepest Decent protocol. Steepest Decent incorporates multiple sequential cycles of conjugate gradient energy minimization against a probability density function that applies spatial constraints derived from the original template and from amino acid-specific properties [43].

### PDBePISA analysis

The Protein Database (PDB) online tool “Proteins, interfaces, structures and assemblies” (PISA) [44] was utilized to calculate the number of hydrogen bonds, salt bridges and disulfide bonds formed between the original and mutated CD4-bound YU2 gp120 containing V3 and the CCR5 N-terminal peptide. PISA is available at the European Bioinformatics Institute website; http://www.ebi.ac.uk/pdbe/prot_int/pistart.html.

## Results and Discussion

### Signature pattern analyses identify multiple Env amino acid alterations outside the V3 loop that are associated with coreceptor specificity

The vast majority of currently available genotypic algorithms have been developed using training sets composed of predominantly B-HIV V3 sequences. To investigate extended Env regions that might further inform these algorithms, we analyzed all full-length, phenotypically characterized patient-derived B-HIV Env sequences available in the Los Alamos HIV Database. Specifically, we analyzed 43 CXCR4-using (23 R5X4 and 20 X4) and 223 R5 B-HIV Env sequences (one sequence per patient) for amino acid alterations that distinguish CXCR4-using from R5 B-HIV Envs. We first performed VESPA to identify positions within CXCR4-using and R5 Envs that have differing consensus or “signature” amino acids. Analysis of V1, V2, V4 and V5 regions was not possible due to significant variations in length that prevented sequence alignment. We compared the type and frequency of all amino acid alterations that differentiate CXCR4-using and R5 Envs, noting those that occurred in ≥5% of CXCR4-using and/or R5 B-HIV strains (Table 1).

**Table 1:** VESPA analysis of R5 and CXCR4-using B-HIV Envs.

Env	Amino	Phenotype		Fisher's exact	Env	Amino	Phenotype		Fisher's exact
position	Acid	CXCR4-using	R5	test (p-value)	position	Acid	CXCR4-using	R5	test (p-value)
**280**	**Asp**	53.5	42.4	ns		**Ile**	24.5	28.3	ns
**(C2)**	**Asn**	46.5	54	ns	**535**	**Leu**	22.4	20.5	ns
**306**	**Gly**	13.6	22.8	ns	**(gp41)**	**Met**	32.7	25.6	ns
**(V3)**	**Arg**	45.5	0.9	<0.0001		**Val**	18.4	25.1	ns
	**Ser**	31.8	75.9	<0.0001		**Asp**	20.4	26.9	ns
**319**	**Ala**	22.7	61.2	<0.0001		**Glu**	16.3	22.4	ns
**(V3)**	**Thr**	61.4	28.1	<0.0001	**621**	**Lys**	6.1	9.6	ns
	–	6.8	8.9	ns	**(gp41)**	**Met**	6.1	0.5	0.0289
	**Asp**	11.4	37.9	<0.0001		**Gln**	26.5	16.9	ns
	**Glu**	22.7	38.8	0.0214		**Ser**	6.1	0.9	ns
**322**	**Lys**	15.9	1.8	0.0008		**Tyr**	10.2	8.2	ns
**(V3)**	**Asn**	6.8	0.9	ns	**720**	**His**	44.9	43.8	ns
	**Gln**	29.5	9.4	0.0003	**(gp41)**	**Leu**	16.3	11.9	ns
	**Arg**	6.1	0.5	0.0289		**Arg**	38.8	44.3	ns
	**Ala**	37.2	32.6	ns		**Asp**	34.7	37.9	ns
**336**	**Glu**	4.7	8.9	ns		**Gly**	0.0	5.0	ns
**(C3)**	**Lys**	4.7	7.6	ns	**750**	**His**	14.3	17.8	ns
	**Thr**	34.9	34.4	ns	**(gp41)**	**Asn**	42.9	21.9	0.0024
	**Val**	4.7	5.4	ns		**Thr**	4.1	8.7	ns
	**Ala**	4.7	7.1	ns		**Asp**	44.9	38.8	ns
	**Asp**	7	0	0.0140	**758**	**Glu**	12.2	6.4	ns
	**Glu**	16.3	4	0.0081	**(gp41)**	**Ile**	4.1	6.4	ns
**440**	**Lys**	11.6	12.6	ns		**Val**	34.7	43.8	ns
**(C4)**	**Gln**	7	6.3	ns		**Cys**	20.4	17.4	ns
	**Arg**	14	48.2	<0.0001	**837**	**Phe**	20.4	32.4	ns
	**Ser**	25.6	19.6	ns	**(gp41)**	**Gly**	24.5	25.6	ns
						**Tyr**	28.6	14.6	0.0059

Amino acid positions are relative to the HXB2 Env. Values represent the percentage of all R5 or CXCR4-using Envs with the indicated amino acid. 43 CXCR4-using (23 R5X4 and 20 X4) and 223 R5 B-HIV Envs were analyzed. ns, not significant. -, deleted sequence.

Nine residues outside V3 displayed signature amino acids that differentiated CXCR4-using and R5 B-HIV Envs, three in gp120 and six in gp41. Statistically significant amino acid alterations occurred at position 440 in gp120 and positions 621, 750 and 837 in gp41. Notably, there was a consistent charge-dependent relationship between amino acids that were present at position 440 in the gp120 C4 region and position 322 in the V3 region that was associated with coreceptor preference. Positively charged Arg/Lys322 and negatively charged Asp/Glu440 occurred more frequently in CXCR4-using Envs than in R5 Envs (Arg/Lys322 in 22% of CXCR4-using and 2.3% of R5 Envs; Asp/Glu440 in 23.3% of CXCR4-using and 4% of R5 Envs). Conversely, negatively charged Asp/Glu322 and positively charged Arg440 occurred more frequently in R5 Envs than in CXCR4-using Envs (Asp/Glu322 in 78.5% of R5 and 34.1% of CXCR4-using Envs; Arg440 in 48.2% of R5 and 14% of CXCR4-using Envs). The associations between Asp/Glu440 frequency and CXCR4 usage, and Arg440 frequency with R5 phenotype was confirmed using a larger independent panel of phenotypically characterized partial Env sequences in the Los Alamos HIV Database that contained at least position 440; 32 CXCR4-using (23 R5X4 and 9 X4) and 70 R5 Envs (one sequence per patient) (Table S1). Although statistically significant amino acid alterations were observed outside V3 at positions 621, 750 and 837 in gp41, we focused our investigation primarily on position 440 in C4, based on the results of a recent study, which showed that inclusion of gp41 mutations does not substantially improve the predictive accuracy of V3 sequence-based coreceptor usage prediction algorithms [45].

### Association between covariance of charged amino acids at Env positions 322/440 and coreceptor specificity of HIV-1 subtype B

An association between charged amino acids at positions 322/440 has been described in a previous study by Rosen et al., which demonstrated a strong preference for positively charged amino acids at position 440 in B-HIV Envs when the amino acid at position 322 was negatively charged [46]. Furthermore, using a relatively small panel of 21 phenotypically characterised B-HIV sequences (10 CXCR4-using and 11 R5), the authors suggest a possible preference for negatively charged amino acids at position 322 and positively charged amino acids at position 440 in R5 B-HIV strains. Here, we sought to determine the significance of these observations by comprehensively characterizing the covariance of charged amino acids at positions 322/440 in 368 phenotypically characterized B-HIV sequences; 75 CXCR4-using (46 R5X4 and 29 X4) and 293 R5 Envs (one sequence per patient).

**Figure 1:**
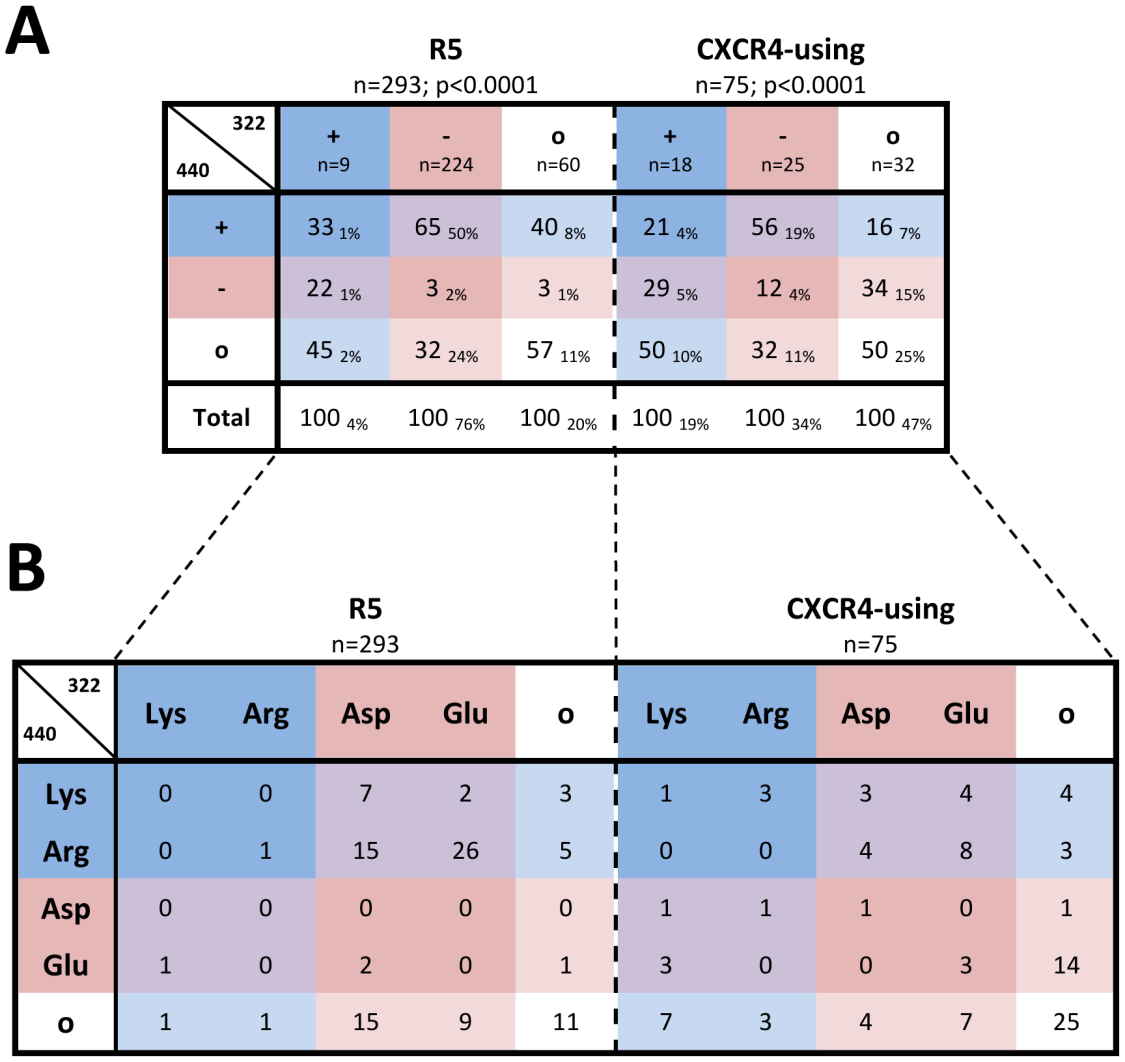
Correlation between charged amino acids at Env positions 322/440 in phenotypically characterized B-HIV strains. (A) Values represent the percentage of Envs that have the indicated type of amino acid (+, positively charged; -, negatively charged; o, neutral) at position 440 out of all Envs with the indicated type of amino acid at position 322. Subscript values represent the percentage of R5 or CXCR4-using Envs that have the indicated combination of amino acid types at positions 322/440. (B) Values represent the percentage of R5 or CXCR4-using Envs that have the combination of indicated amino acids at Env positions 322/440. Amino acids are colored according to charge; positively charged (+, Arg and Lys) in blue, negatively charged (-, Asp and Glu) in red and neutral (o) in white. 322/440 amino acid combinations are colored according to charge; +322/+440 in dark blue, −322/−440 in dark red, +322/−440 or −322/+440 in purple, +322/o440 or o322/+440 in light blue, −322/o440 or o322/−440 in light red and o322/o440 in white. P-values were calculated by chi-square analysis.

Chi-square analysis revealed a statistically significant covariant association between amino acid charge at positions 322/440 for both CXCR4-using and R5 Envs (p<0.0001) (Figure 1A). For ease of communication, we hereafter use +/−/o to designate a positively charged, negatively charged or neutral amino acid at positions 322/440, respectively. We found that 76% of R5 Envs were -322 (of which 65% were +440, 32% o440 and 3% −440), 20% were o322 (of which 57% were o440, 40% +440 and 3% −440) and only 4% were +322 (of which 45% were o440, 33% +440 and 22% −440). For CXCR4-using Envs, 47% were o322 (of which 50% were o440, 34% −440 and 16% +440), 34% were −322 (of which 56% were +440, 32% o440 and 12% −440) and 19% were +322 (of which 50% were o440, 29% −440 and 21% +440). Overall, R5 Envs displayed a preference for −322/+440, particularly Glu322/Arg440 (26%; Figure 1B), and CXCR4-using Envs most frequently displayed o322/o440 (25%). Together, these analyses show that R5 and CXCR4-using Envs frequently exhibit different 322/440 amino acid charge profiles, suggesting that (i) charge clashes between these positions are unfavorable, and (ii) that mutations at these positions may contribute to coreceptor specificity.

### Analysis of nucleotide codon usage at position 440 of B-HIV Envs suggests that position 440 mutations are driven by the presence of charged amino acids at position 322

We next sought to better understand the molecular mechanisms underlying the linkage between charge covariance at positions 322/440 and coreceptor specificity of B-HIV strains. Yamaguchi-Kabata et al. [47] has suggested that mutations within V3 that are associated with B-HIV coreceptor usage may affect the mutation rate at residues outside V3 in a charge-dependent manner. While some mutagenesis studies support a role for amino acids at position 440 for determining coreceptor usage [34], [37], [48], the majority of studies have demonstrated that mutations in V3 alone are often sufficient for a complete switch from CCR5- to CXCR4-usage [22], [26], [36], [37], [49]. Considering these findings and given that distinct charge covariance patterns are preferred among R5 and CXCR4-using Envs (−322/+440 and o322/o440, respectively; Figure 1A), we hypothesized that mutations at position 440 may be influenced by the presence of charged amino acids at position 322, which is a key V3 determinant of coreceptor usage. If this were the case we would expect that, where there is a shift away from a preferred charged amino acid at position 440, which compliments the presence of a charged amino acid at position 322, the nucleotide codon encoding the suboptimal amino acid at position 440 would be within one nucleotide substitution from the codon encoding the preferred amino acid.

**Figure 2:**
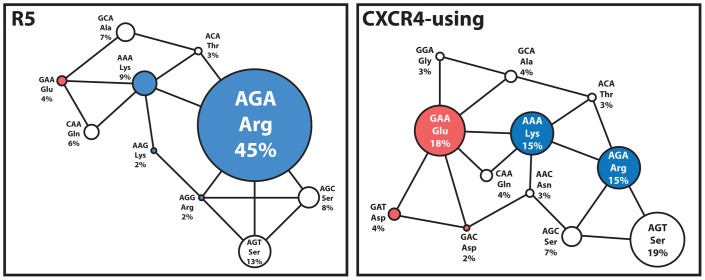
Nucleotide codon usage of R5 and CXCR4-using B-HIV Envs at position 440. The size of each node indicates the relative frequency of the indicated nucleotide codon. A black line connects codons separated by one nucleotide substitution. Codons encoding positively charged amino acids at colored blue, codons encoding negatively charged amino acids are colored red and codons encoding neutral amino acids are colored white. Nucleotide codons at position 440 were aligned and analysed using CLC Main Workbench version 6.5. 75 CXCR4-using (46 R5X4 and 29 X4) and 293 R5 sequences were analyzed.

Our analyses suggest that this may indeed be the case among patient-derived B-HIV sequences in the Los Alamos HIV Database; 75 CXCR4-using (46 R5X4 and 29 X4) and 293 R5 Envs (one sequence per patient). In R5 Envs, 45% used the AGA codon that encodes the preferred Arg440 (Figure 2), while the majority of Envs that did not express the AGA codon used codons within one nucleotide substitution of AGA (67%). Thus the distribution of nucleotide codons among R5 B-HIV Envs does not appear to be random, exhibiting a drive towards the AGA codon that encodes the preferred amino acid Arg440. For instance, while there are six possible Ser codons, only those within one nucleotide substitution of AGA (Arg) were utilized, namely AGC and AGT. These results are concordant with those of previous studies [47]. In comparison, among CXCR4-using Envs there was an increase in the frequency of nucleotide codons encoding the preferred negatively charged amino acids Glu/Asp440 and neutral amino acids o440. Notably, CXCR4-using Envs used the nucleotide codon GAA that encodes the amino acid Glu (GAA; 18%) more frequently than the GAT or GAC codons that encode Asp (GAT; 4% and GAC; 2%) (Figure 2). This may be due to the fact that the nucleotide codon GAA is closer in terms of number of substitutions to the most commonly utilized R5 Env nucleotide codon AGA, and thus would likely emerge more frequently in CXCR4-using Envs during B-HIV disease progression as R5 viruses gain the ability to use CXCR4. Interestingly, although the frequency of the nucleotide codon AGA for positively charged Arg440 dramatically decreased in CXCR4-using Envs (15%) compared to R5 Envs (45%), the AAA codon for similarly positively charged Lys slightly increased in CXCR4-using Envs (15%) compared to R5 Envs (9%) (Figure 2). Notably, this codon (AAA) is one nucleotide substitution from both the GAA nucleotide codon preferred by CXCR4-using Envs and the AGA nucleotide codon preferred by R5 Envs. Thus, the nucleotide codon AAA may act as an intermediary between R5 and CXCR4-using Envs, which may also explain why the presence of positively charged Lys440 did not differentiate R5 and CXCR4-using Envs (Table 1).

### Structural modeling suggests an electrostatic interaction between charged amino acids at B-HIV Env positions 322/440 and charged amino acids in the CCR5 N-terminus

**Figure 3:**
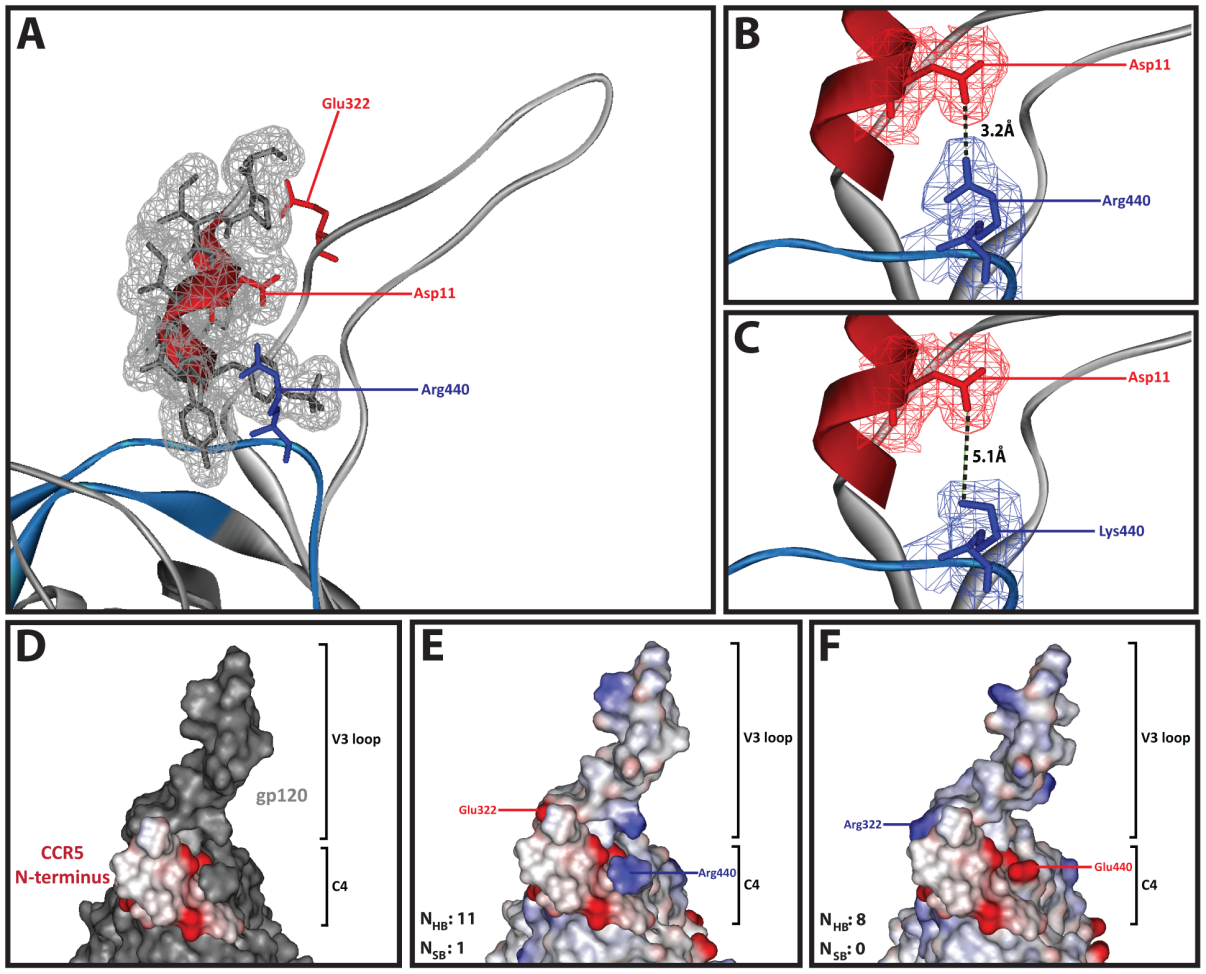
Three-dimensional model of the CD4-bound gp120 containing V3 and C4 docked to a CCR5 N-terminus peptide. (A) The gp120 molecule is shown in ribbon representation, the V3 is shown in grey and the C4 region is shown in blue. The CCR5 N-terminus peptide is shown in ribbon representation (red) with wire mesh van der Waals surface representation, and the amino acids are shown in stick representation. Asp11 in the CCR5 N-terminus and Glu322 and Arg440 in gp120 are shown in stick representation and colored according to charge; positively charged residues are colored blue and negatively charged residues colored red. (B, C) Close up of gp120 and the CCR5 N-terminus peptide interface is represented as described above. Notably, CCR5 N-terminus mesh surface representation and amino acid stick representation was removed, and mesh surface van der Waals surface representation was added to Asp11 in the CCR5 N-terminus, Arg440 (B) and Lys 440 (C). (D) Three-dimensional model of CD4-bound gp120 containing V3 and C4 docked to the CCR5 N-terminus (to aid visual distinction between gp120 and the CCR5 N-terminus peptide) with (E) original Glu322/Arg440 genotype or (F) mutated Arg322/Glu440 genotype, shown in surface charge representation colored according to charge. The number of hydrogen bonds (N_HB_) and salt bridges (N_SB_) predicted to form at the interface between gp120 and the CCR5 N-terminus was calculated using PISA software.

In order to contextualize the linkage between B-HIV Env positions 322/440 and coreceptor specificity, we generated homology models based on the crystal structure of the CD4-bound YU2 gp120 containing V3 and C4 docked with a sulfated CCR5 N-terminus peptide (amino acids 7 through 15) [20]. Amino acids at positions 322/440 are located in close proximity to the CCR5 N-terminus peptide, which binds between the base of the V3 loop (grey ribbon) and C4 region (blue ribbon) (Figure 3A). The amino acid at position 440 likely interacts with Asp11 in the CCR5 N-terminus. Figure 3B shows Arg440 positioned within 3.2Å of Asp11, whereas figure 3C shows Lys440 positioned 5.1Å from Asp11, suggesting that a stronger electrostatic interaction may exist between gp120 and the CCR5 N-terminus when Arg440 is present, which, in addition to our codon usage analysis, could potentially explain why there is a reduced preference for Lys440 in R5 B-HIV strains (Table 1). In order to visualize the electrostatic binding interface between gp120 and the CCR5 N-terminus peptide, we next rendered the protein structure according to surface charge of the amino acid residues. Figure 3D was included to aid visual distinction between gp120 and the CCR5 N-terminus peptide. In concordance with a previous study [50], we found that Arg440 closely associates with Asp11 and sulfotyrosines at positions 10 and 14 in CCR5 (Figure 3E), suggesting that these residues form an electrostatic binding interface between CCR5 and gp120. When compared to a mutated molecular model containing an inverse Arg322/Glu440 genotype, there is a potential charge clash between Glu440 and the CCR5 N-terminus (Figure 3F). Analysis of the binding surface using the PISA tool revealed that 11 hydrogen bonds and 1 salt bridge potentially connect Glu322/Arg440 gp120 and the CCR5 N-terminus, of which 3 hydrogen bonds and the salt bridge are lost in the inverse Arg322/Glu440 model. This may explain the preference of R5 B-HIV strains for Arg440 in gp120, such that it can interact with multiple negatively charged residues in the CCR5 N-terminus to enhance coreceptor binding.

Previous studies describing correlations between charged amino acids at positions 322/440 have suggested that a direct interaction may occur between amino acids at 322/440, yet amino acids at position 440 do not bind directly to the coreceptor [46], [47], [51], [52]. However, Cimbro et al. [53] recently demonstrated that intra-molecular interactions occur in CD4-unbound Envs that are mimicked by intermolecular interactions between gp120 and CCR5 after CD4 binding. Thus we propose a similar mechanism whereby amino acids at positions 322 and 440 could potentially interact directly with one another in CD4-unbound Envs, but upon CD4 binding they could each interact with charged residues in the CCR5 N-terminus. Future *in vitro* mutagenesis studies exploring the ability of various 322/440 Env genotypes to bind N-terminal CCR5 peptides in the presence and absence of CD4 will be required to test this hypothesis.

### Position 440-based parameters may improve the performance of V3 sequence-based algorithms for predicting the coreceptor usage of B-HIV strains

We next assessed whether the inclusion of position 440-based prediction criteria into commonly used V3 sequence-based genotypic algorithms [“11/25 rule”, geno2pheno (G2P) and WebPSSM] improves their accuracy for predicting B-HIV coreceptor usage, relative to gold-standard phenotypic cell entry assay results. Specifically, the algorithms were modified to include a final criterion “440 rule” for predicting CXCR4-usage, such that all sequences predicted by the unmodified genotypic algorithms to be R5 were rescreened and predicted to be CXCR4-using if Asp or Glu were present at position 440. Initially, the modified algorithms were tested against the panel of phenotypically characterized full-length B-HIV Envs that were used to design the “440 rule”, i.e. 43 CXCR4-using (23 R5X4 and 20 X4) and 223 R5 (one sequence per patient) (Table 1). Table 2 presents the sensitivity and specificity (measured against phenotypic cell entry assay results), as well as the area under the receiver operating characteristic curve (AUROC), for the original unmodified algorithms and the modified versions that include the “440 rule”. When tested against the “design sequence set”, incorporation of the “440 rule” improved sensitivity with minimal cost to specificity for the “11/25 rule” (+9% sensitivity; −3.1% specificity), G2P at all false positive rates (+7% to +9.3% sensitivity; −2.2% to −4% specificity) and both WebPSSM algorithms (+7% sensitivity; −3.1% to −3.4% specificity), however the resultant increases in AUROC were not found to be statistically significant compared to the unmodified algorithms.

**Table 2:** Performance of genotypic algorithms modified to include the “440 rule”.

Genotypic	Design Sequence Set			Independent Test Sequence Set		
algorithm	% Sens	% Spec	AUROC	% Sens	% Spec	AUROC
**440 rule**	23.1	96	0.60	39.4	95.5	0.67
**11/25 rule**	65.1 (+9.3)	99.6 (-3.1)	0.8 (+0.03; p = 0.30)	33.3 (+24.3)	98.5 (−4.5)	0.66 (+0.10; p = 0.11)
**G2P FPR 1%**	55.8 (+9.3)	99.6 (−4.1)	0.78 (+0.03; p = 0.33)	33.3 (+24.3)	100 (−4.5)	0.58 (+0.18; ***p = 0.01***)
**G2P FPR 2.5%**	74.4 (+7)	98.2 (−4)	0.86 (+0.02; p = 0.38)	51.5 (+15.2)	100 (−4.5)	0.63 (+0.18; ***p = 0.01***)
**G2P FPR 5%**	79.1 (+6.9)	94.2 (−3.6)	0.87 (+0.01; p = 0.37)	60.6 (+15.2)	100 (−4.5)	0.65 (+0.21; ***p = 0.003***)
**G2P FPR 5.75%**	81.4 (+7)	92.1 (−3.6)	0.87 (+0.02; p = 0.37)	60.6 (+15.2)	100 (−4.5)	0.65 (+0.21; ***p = 0.003***)
**G2P FPR 10%**	81.4 (+7)	86.2 (−3.6)	0.84 (+0.02; p = 0.38)	69.7 (+9.1)	91 (−4.4)	0.80 (+0.02; p = 0.37)
**G2P FPR 15%**	81.4 (+7)	82.1 (−3.1)	0.82 (+0.02; p = 0.37)	69.7 (+9.1)	85.1 (−4.5)	0.77 (+0.02; p = 0.43)
**G2P FPR 20%**	83.7 (+7)	70.5 (−2.2)	0.77 (+0.02; p = 0.35)	72.7 (+9.1)	77.6 (−4.5)	0.75 (+0.02; p = 0.38)
**WebPSSM_X4R5_**	74.4 (+7)	98.2 (−3.6)	0.86 (+0.02; p = 0.74)	48.5 (+15.1)	98.5 (−4.5)	0.74 (+0.05; p = 0.25)
**WebPSSM_SI/NSI_**	76.7 (+7)	94.2 (−3.1)	0.85 (+0.02; p = 0.71)	48.5 (+15.1)	97 (−4.5)	0.73 (+0.05; p = 0.25)

The “440 rule” involves rescreening sequences predicted to be R5 by the indicated genotypic algorithm for the presence of Asp or Glu at position 440, the presence of which results in a “CXCR4-using” prediction. % Sens, sensitivity was calculated by dividing the number of correctly predicted CXCR4-using sequences by the total number of phenotypically characterised CXCR4-using sequences. % Spec, specificity was calculated by dividing the number of correctly predicted R5 sequences by the number of phenotypically characterised R5 sequences. Values in parentheses represent percentage differences between modified and unmodified algorithms. Differences in area under the receiver operating characteristic curve (AUROC) considered significant (p≤0.05) are highlighted in bold and italicized. 43 CXCR4-using (23 R5X4 and 20 X4) and 223 R5 B-HIV design sequences and 32 CXCR4-using (23 R5X4 and 9 X4) and 70 R5 B-HIV test sequences were analysed. FPR, false positive rate.

To account for any bias that may have been introduced through testing the modified algorithms against the same sequences used to develop the “440 rule”, we next tested their performance using an independent panel of phenotypically characterized, partially-sequenced Envs in the Los Alamos HIV Database containing at least V3 and position 440; 32 CXCR4-using (23 R5X4 and 9 X4) and 70 R5 (one sequence per patient). When tested against this independent sequence panel, incorporation of the “440 rule” again improved sensitivity with minimal cost to specificity for the “11/25 rule” (+24.2% sensitivity, −4.5% specificity), G2P at all false positive rates (+9.1 to +24.2% sensitivity, −4.4 to −4.5% specificity) and both WebPSSM algorithms (+15.2% sensitivity, −4.5% specificity), and significantly improved the AUROC of G2P at FPRs of 1% (p = 0.013), 2.5% (p = 0.011), 5% (p = 0.003) and 5.75% (p = 0.003), the latter of which is the recommended G2P cutoff as determined by retrospective analysis of Pfizer MVC clinical trials MOTIVATE-1, -2 and A4001029 [30], [54]. Although additional analyses are required to determine whether incorporation of the “440 rule” into genotypic algorithms improves their capacity to predict treatment outcomes for patients taking CCR5 antagonists, our findings demonstrate that inclusion of the “440 rule” improves V3 sequence-based coreceptor usage predictions relative to phenotypic cell entry assays.

Incidentally, in concordance with the aforementioned study by Thielen et al. [45], which showed that the addition of mutations in gp41 does no improve the predictive accuracy of genotypic algorithms, we found that genotypic algorithms were not improved by the inclusion of prediction parameters based on mutations at positions 621, 750 and 837 in gp41 that we showed (1) correlate with B-HIV coreceptor usage (Table S2).

### Covariance of amino acids at Env positions 322/440 does not exhibit a clear association with coreceptor usage in non-B HIV-1 subtypes

**Figure 4:**
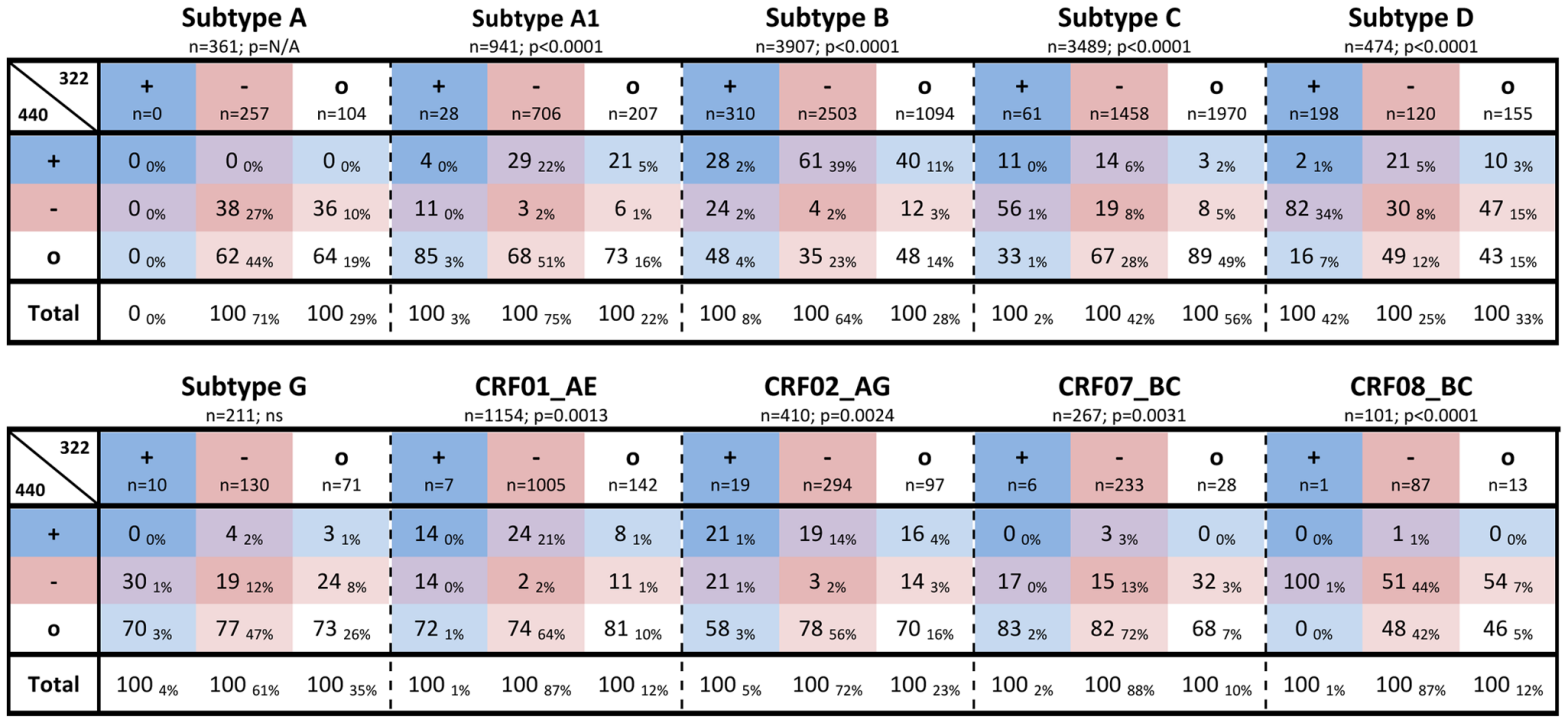
Analysis of covariance of charged amino acids at Env positions 322/440 in non-B subtype HIV-1 strains. Values represent the percentage of Envs within each subtype that have the indicated type of amino acid (+, positively charged; -, negatively charged; o, neutral) at position 440 out of all Envs with the indicated type of amino acid at position 322. Subscript values represent the percentage of Envs within each subtype that have the indicated combination of amino acid types at positions 322/440. Amino acids are colored according to charge, as described in the legend for Figure 1. P-values were calculated by chi-square analysis (ns, not significant; N/A, not applicable).

Previous studies have suggested that covariance between charged amino acids at positions 322 and 440 does not occur in HIV-1 subtypes A (A-HIV) or C (C-HIV) [46], [47]. To expand on these studies we next characterized 322/440 covariance in subtypes A, A1, C, D (D-HIV), G, CRF01_AE (AE-HIV), CRF02_AG, CRF07_BC and CRF08_BC using all phenotypically-characterized sequences available in the Los Alamos HIV Database, as well as a panel of C-HIV Envs recently characterized by our laboratory (one sequence per patient) [26]. The low frequency of Envs with charge clashes at positions 322/440 (+322/+440 or −322/−440) suggests that covariance may exist in HIV-1 subtypes A1, C, D, CRF01_AE and CRF02_AG (Figure 4).

**Figure 5:**
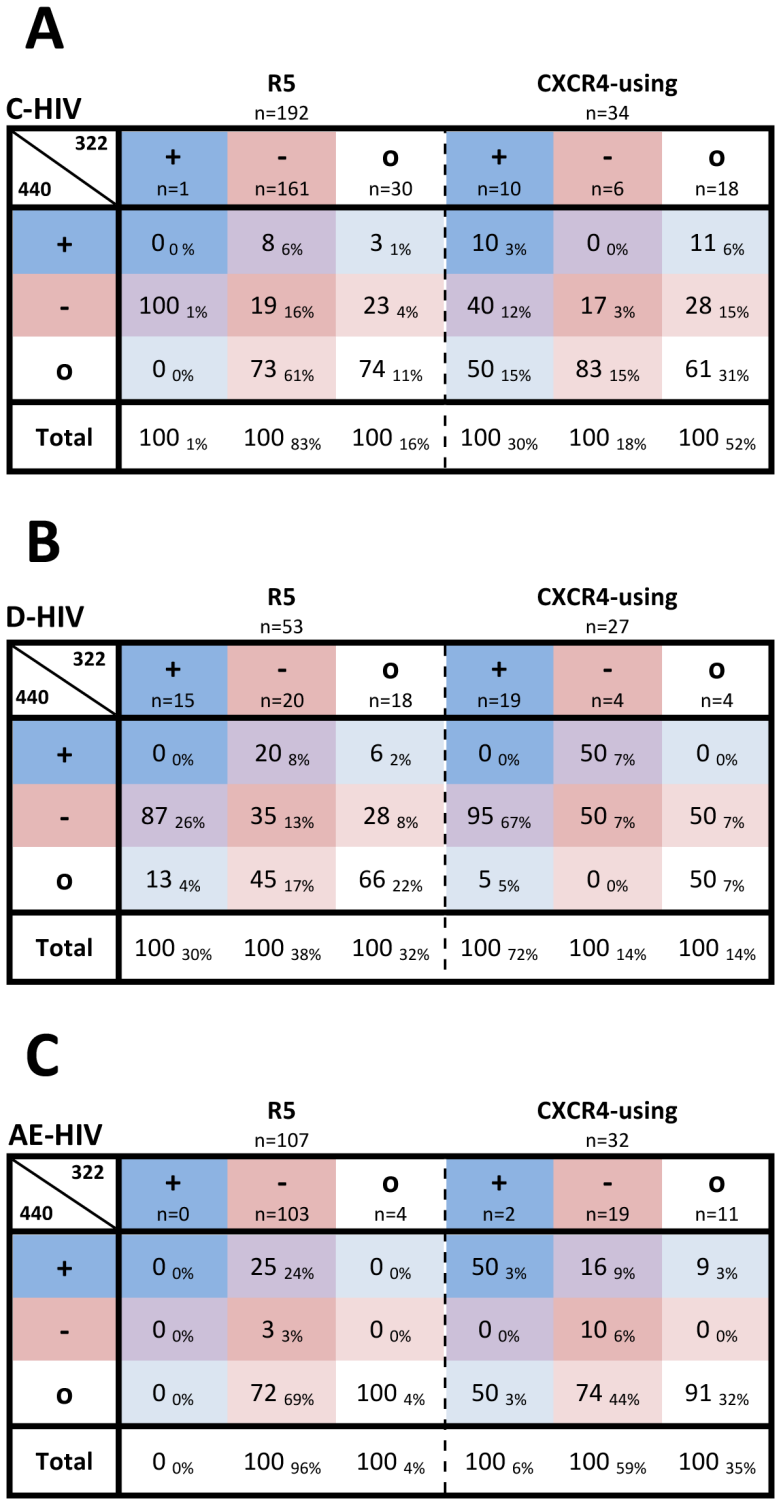
Correlation between charged amino acids at Env positions 322/440 and coreceptor usage for phenotypically characterized C-HIV, D-HIV and AE-HIV strains. Values represent the percentage of R5 or CXCR4-using Envs within C-HIV (A), D-HIV (B) and AE-HIV (C) subtypes that have the indicated type of amino acid (+, positively charged; −, negatively charged; o, neutral) at position 440 out of all Envs with the indicated type of amino acid at position 322. Subscript values represent the percentage of R5 or CXCR4-using Envs within each subtype that have the indicated combination of amino acid types at positions 322/440. Amino acids are colored according to charge, as described in the legend for Figure 1.

We next investigated whether these potentially covariant sites influence coreceptor usage of non-B HIV-1 subtypes. Unfortunately, the paucity of phenotypically characterized sequences containing positions 322/440 in non-B HIV-1 subtypes limited our analysis to C-, D- and AE-HIV strains (Figure 5). Unlike B-HIV, we found that C-HIV R5 Envs have preference for a -322/o440 genotype (61%), yet similar to B-HIV, emergence of CXCR4-using C-HIV Envs was associated with a preference for o322/o440 (31%). Covariance at 322/440 in D-HIV was not associated with coreceptor usage, as both the majority of R5 and CXCR4-using D-HIV sequences exhibited a preference for the same +322/−440 genotype, similar to CXCR4-using B-HIV. However, considering that there was a marked difference in frequency of CXCR4-using D-HIV Envs with a −322/+440 genotype (67%) compared to R5 Envs (26%), these findings suggest that R5 D-HIV viruses may be predisposed for emergence of CXCR4-using viruses, which may explain the high incidence of CXCR4-using strains in the D-HIV epidemic. Similarly, 322/440 preferences within AE-HIV strains could not distinguish R5 from CXCR4-using strains, with both phenotypes exhibiting a relatively high frequency of -322/o440 genotype.

Notably, the performance of genotypic algorithms for predicting the coreceptor usage of C-, D- and AE-HIV strains (relative to phenotypic cell entry assays) was not improved by inclusion of the “440 rule” (Table S3). This may be explained by the fact that (i) a clear covariance pattern was not apparent for these subtypes and (ii) current algorithms, the vast majority of which were designed against B-HIV, often perform poorly against non-B HIV-1 strains because the determinants of coreceptor usage are often subtype specific [41], [55]-[59]. Subsequent VESPA analysis of C-, D- and AE-HIV V3 and C4 Env regions confirmed that the presence of Asp/Glu440, which is the basis of the “440 rule”, does not differentiate CXCR4-using from R5 Envs of these subtypes (data not shown). In future studies we plan to further analyze non-B HIV-1 Envs to identify other potentially subtype specific amino acid alterations outside V3 that are associated with coreceptor usage, which may be included in novel subtype specific algorithms such as our recently developed subtype C specific algorithm CoRSeq_V3-C_ [41].

## Conclusions

Analysis of every available phenotypically characterized B-HIV Env sequence in the Los Alamos HIV Database revealed a highly significant association between covariant charged amino acids at Env positions 322/440 and coreceptor usage. Investigation into the mechanisms underlying this linkage suggests that, although amino acid alterations at position 440 alone are unlikely to be a necessary determinant of B-HIV coreceptor usage, the presence of oppositely charged compensatory amino acids at these sites might provide an advantage within a viral population. This advantage may involve stabilizing electrostatic interactions between amino acids at positions 322/440 of CD4-unbound gp120, and between these residues of CD4-bound gp120 and the CCR5 N-terminal region. These findings provide new mechanistic insights into Env determinants outside V3 that contribute to coreceptor usage of HIV-1 subtype B.

We also showed that a position “440 rule” can improve the sensitivity of commonly used genotypic algorithms for detecting CXCR4-usage of B-HIV strains, but not of non-B HIV-1 strains, without substantially compromising specificity, and significantly improves the AUROC of G2P at the recommended FPR of 5.75%. The continued rapid improvement of HIV-1 Env sequencing techniques offers potential for expansion of V3 sequence-based genotypic algorithms to include regions outside V3 such as the position 440, and may potentially permit more reliable predictions of HIV-1 coreceptor usage. Thus, the results of our study may not only inform the optimization of current tools, they may be incorporated into the development of more sophisticated, next-generation coreceptor usage prediction algorithms.

## Supporting Information

Table S1 **Expanded VESPA analysis of B-HIV sequences containing position 440.** Amino acid positions are relative to HXB2 Env. Values represent the percentage of CXCR4-using or R5 Envs with the indicated amino acid. 75 CXCR4-using (46 R5X4 and 29 X4) and 293 R5 B-HIV Env sequences were analyzed. ns, not significant.
(PDF)

Table S2 **Performance of genotypic algorithms modified to include the “440 rule”.** The “440 rule” involves rescreening sequences predicted to be R5 by the indicated genotypic algorithm for the presence of Asp or Glu at position 440, the presence of which results in a “CXCR4-using” prediction. % Sens, sensitivity was calculated by dividing the number of correctly predicted CXCR4-using sequences by the total number of phenotypically characterised CXCR4-using sequences. % Spec, specificity was calculated by dividing the number of correctly predicted R5 sequences by the number of phenotypically characterised R5 sequences. Values in parentheses represent percentage differences between modified and unmodified algorithms. Differences in area under the receiver operating characteristic curve (AUROC) considered significant (p≤0.05) are highlighted in bold and italicized. 34 CXCR4-using (23 R5X4 and 11 X4) and 193 R5 C-HIV sequences, 27 CXCR4-using (18 R5X4 and 9 X4) and 53 R5 D-HIV sequences, and 32 CXCR4-using (13 R5X4 and 19 X4) and 107 R5 AE-HIV sequences were analysed. FPR, false positive rate.
(PDF)

Table S3 **Performance of genotypic algorithms modified to include prediction parameters based on mutations at positions 621, 750 and 837 in gp41.** Prediction parameters based on mutations at positions 621, 750 and 837 in gp41 involve rescreening sequences predicted to be R5 by the indicated genotypic algorithm for the presence of an Met amino acid at position 621, an Asn amino acid at position 750, or a Thr amino acid at position 837, respectively, the presence of which results in a “CXCR4-using” prediction. % Sens, sensitivity was calculated by dividing the number of correctly predicted CXCR4-using sequences by the total number of phenotypically characterised CXCR4-using sequences. % Spec, specificity was calculated by dividing the number of correctly predicted R5 sequences by the number of phenotypically characterised R5 sequences. Values in parentheses represent the percentage difference between modified and unmodified genotypic algorithm. 43 CXCR4-using (23 R5X4 and 20 X4) and 223 R5 B-HIV design sequences were analysed. FPR, false positive rate.
(PDF)
