# Supplementary material for: Covariance of Charged Amino Acids at Positions 322 and 440 of HIV-1 Env Contributes to Coreceptor Specificity of Subtype B Viruses, and Can Be Used to Improve the Performance of V3 Sequence-Based Coreceptor Usage Prediction Algorithms
Source: PLoS One. 2014 Oct 14;9(10):e109771. doi: 10.1371/journal.pone.0109771 (PMC4196930; doi:10.1371/journal.pone.0109771)
Supplement: Table S1 — Expanded VESPA analysis of B-HIV sequences containing position 440. Amino acid positions are relative to HXB2 Env. Values represent the percentage of CXCR4-using or R5 Envs with the indicated amino acid. 75 CXCR4-using (46 R5X4 and 29 X4) and 293 R5 B-HIV Env sequences were analyzed. ns, not significant. (PDF) [file pone.0109771.s001.pdf]

Table S1. Expanded VESPA analysis of B-HIV sequences containing position 440.

Env position	Amino acid	Phenotype		Fisher's exact test (p-value)
		CXCR4-using	R5	
440 (C4)	Ala	6	4.5	ns
	Asp	3	0	ns
	Glu	36.4	4.5	<0.0001
	Lys	12.1	9	ns
	Gln	6.1	4.5	ns
	Arg	18.2	46.3	<0.0001
	Ser	18.2	26.9	ns
	Thr	0	1.5	ns
	-	0	2.8	ns
