# Supplementary material for: Covariance of Charged Amino Acids at Positions 322 and 440 of HIV-1 Env Contributes to Coreceptor Specificity of Subtype B Viruses, and Can Be Used to Improve the Performance of V3 Sequence-Based Coreceptor Usage Prediction Algorithms
Source: PLoS One. 2014 Oct 14;9(10):e109771. doi: 10.1371/journal.pone.0109771 (PMC4196930; doi:10.1371/journal.pone.0109771)
Supplement: Table S2 — Performance of genotypic algorithms modified to include the “440 rule”. The “440 rule” involves rescreening sequences predicted to be R5 by the indicated genotypic algorithm for the presence of Asp or Glu at position 440, the presence of which results in a “CXCR4-using” prediction. % Sens, sensitivity was calculated by dividing the number of correctly predicted CXCR4-using sequences by the total number of phenotypically characterised CXCR4-using sequences. % Spec, specificity was calculated by dividing the number of correctly predicted R5 sequences by the number of phenotypically characterised R5 sequences. Values in parentheses represent percentage differences between modified and unmodified algorithms. Differences in area under the receiver operating characteristic curve (AUROC) considered significant (p≤0.05) are highlighted in bold and italicized. 34 CXCR4-using (23 R5X4 and 11 X4) and 193 R5 C-HIV sequences, 27 CXCR4-using (18 R5X4 and 9 X4) and 53 R5 D-HIV sequences, and 32 CXCR4-using (13 R5X4 and 19 X4) and 107 R5 AE-HIV sequences were analysed. FPR, false positive rate. (PDF) [file pone.0109771.s002.pdf]

Table S3. Performance of genotypic algorithms modified to include prediction parameters based on mutations at positions 621, 750 and 837 in gp41.

Genotypic Algorithms	Position 621			Position 750			Position 837		
	Sens	Spec	AUROC	Sens	Spec	AUROC	Sens	Spec	AUROC
**11/25 rule**	70.7 (2.4)	94.2 (-0.5)	0.82 (+0.01; p=0.44)	78 (+9.7)	73 (-21.7)	0.76 (-0.06; p=0.17)	75.6 (+7.3)	80.4 (-14.3)	0.78 (-0.03; p=0.29)
**G2P FPR 1%**	78 (2.4)	97.4 (-0.5)	0.88 (+0.01; p=0.43)	82.9 (+7.3)	75.1 (-22.8)	0.79 (-0.08; p=0.09)	80.5 (+4.9)	83.6 (-14.3)	0.82 (-0.05; p=0.20)
**G2P FPR 2.5%**	80.5 (2.5)	92.6 (-0.5)	0.87 (+0.01; p=0.43)	85.3 (+7.4)	70.9 (-22.2)	0.78 (-0.07; p=0.10)	82.9 (+4.9)	78.8 (-14.3)	0.81 (-0.05; p=0.21)
**G2P FPR 5%**	61 (2.5)	98.9 (-0.6)	0.80 (+0.01; p=0.44)	70.7 (+12.2)	76.2 (-23.3)	0.73 (-0.06; p=0.20)	68.3 (+9.8)	84.7 (-14.8)	0.76 (-0.03; p=0.35)
**G2P FPR 5.75%**	80.5 (2.5)	97.4 (-0.5)	0.89 (+0.01; p=0.42)	87.8 (+9.8)	75.1 (-22.8)	0.81 (-0.06; p=0.12)	82.9 (+4.9)	83.6 (-14.3)	0.83 (-0.05; p=0.19)
**G2P FPR 10%**	82.9 (0)	92.6 (-0.5)	0.88 (-0.003; p=0.48)	87.8 (+4.9)	71.4 (-21.7)	0.80 (-0.08; p=0.07)	87.8 (+4.9)	78.8 (-14.3)	0.83 (-0.05; p=0.19)
**G2P FPR 15%**	85.4 (0)	91.5 (-0.6)	0.88 (-0.003; p=0.48)	90.2 (+4.8)	70.9 (-21.2)	0.81 (-0.08; p=0.07)	90.2 (+4.8)	77.8 (-14.3)	0.84 (-0.05; p=0.19)
**G2P FPR 20%**	85.4 (0)	84.7 (-0.5)	0.85 (-0.003; p=0.48)	90.2 (+4.8)	65.1 (-20.1)	0.78 (-0.08; p=0.10)	90.2 (+4.8)	71.4 (-13.8)	0.81 (-0.05; p=0.22)
**$WebPSSM_{X4R5}$**	85.4 (0)	80.4 (-0.6)	0.83 (-0.003; p=0.48)	90.2 (+4.8)	62.4 (-18.6)	0.76 (-0.07; p=0.13)	90.2 (+4.8)	67.7 (-13.3)	0.79 (-0.04; p=0.24)
**$WebPSSM_{SI/NSI}$**	87.8 (0)	68.3 (-0.5)	0.78 (-0.003; p=0.48)	92.7 (+4.9)	52.9 (-15.9)	0.73 (-0.06; p=0.20)	92.7 (+4.9)	58.2 (-10.6)	0.75 (-0.03; p=0.33)
