# Supplementary material for: Covariance of Charged Amino Acids at Positions 322 and 440 of HIV-1 Env Contributes to Coreceptor Specificity of Subtype B Viruses, and Can Be Used to Improve the Performance of V3 Sequence-Based Coreceptor Usage Prediction Algorithms
Source: PLoS One. 2014 Oct 14;9(10):e109771. doi: 10.1371/journal.pone.0109771 (PMC4196930; doi:10.1371/journal.pone.0109771)
Supplement: Table S3 — Performance of genotypic algorithms modified to include prediction parameters based on mutations at positions 621, 750 and 837 in gp41. Prediction parameters based on mutations at positions 621, 750 and 837 in gp41 involve rescreening sequences predicted to be R5 by the indicated genotypic algorithm for the presence of an Met amino acid at position 621, an Asn amino acid at position 750, or a Thr amino acid at position 837, respectively, the presence of which results in a “CXCR4-using” prediction. % Sens, sensitivity was calculated by dividing the number of correctly predicted CXCR4-using sequences by the total number of phenotypically characterised CXCR4-using sequences. % Spec, specificity was calculated by dividing the number of correctly predicted R5 sequences by the number of phenotypically characterised R5 sequences. Values in parentheses represent the percentage difference between modified and unmodified genotypic algorithm. 43 CXCR4-using (23 R5X4 and 20 X4) and 223 R5 B-HIV design sequences were analysed. FPR, false positive rate. (PDF) [file pone.0109771.s003.pdf]

Table S2. Performance of genotypic algorithms modified to include the "440 rule".

Genotypic algorithm	C-HIV			D-HIV			AE-HIV		
	% Sens	% Spec	AUROC	% Sens	% Spec	AUROC	% Sens	% Spec	AUROC
**11/25 rule**	41.2 (+14.7)	99 (-19)	0.70 (-0.02; p=0.39)	71.4 (+13.2)	71.7 (-22.6)	0.72 (-0.05; p=0.30)	65.6 (+6.3)	99.1 (-2.8)	0.82 (0.02; p=0.41)
**G2P FPR 1%**	61.8 (+2.9)	99.5 (-19.5)	0.81 (-0.08; p=0.12)	38.5 (+47.2)	98.1 (-45.3)	0.68 (0.01; p=0.46)	81.3 (+3.1)	99.1 (-2.8)	0.90 (0.001; p=0.49)
**G2P FPR 2.5%**	73.5 (0)	99.5 (-19.5)	0.87 (-0.1; p=0.07)	69.2 (+19.3)	88.7 (-35.9)	0.79 (-0.08; p=0.17)	93.8 (0)	92.5 (-1.8)	0.93 (-0.01; p=0.43)
**G2P FPR 5%**	79.4 (0)	97.9 (-19.4)	0.89 (-0.1; p=0.06)	80.8 (+15.4)	69.8 (-24.5)	0.75 (-0.05; p=0.30)	93.8 (0)	80.4 (-1)	0.87 (-0.01; p=0.47)
**G2P FPR 5.75%**	82.4 (0)	97.9 (-19.4)	0.90 (-0.1; p=0.05)	80.8 (+15.4)	67.9 (-24.5)	0.74 (-0.05; p=0.61)	93.8 (0)	77.6 (-1)	0.86 (-0.01; p=0.47)
**G2P FPR 10%**	82.4 (0)	95.4 (-19.5)	0.89 (-0.1; p=0.06)	92.3 (+3.9)	58.5 (-18.9)	0.75 (-0.08; p=0.20)	96.9 (0)	56.1 (0)	0.77 (0)
**G2P FPR 15%**	85.3 (0)	93.3 (-18.9)	0.89 (-0.09; p=0.06)	92.3 (+3.9)	56.6 (-17)	0.74 (-0.07; p=0.23)	96.9 (0)	48.6 (0)	0.73 (0)
**G2P FPR 20%**	85.3 (0)	89.2 (-18.9)	0.87 (-0.09; p=0.07)	92.3 (+3.9)	49.1 (-17)	0.71 (-0.07; p=0.24)	96.9 (0)	40.2 (0)	0.69 (0; p=0.5)
**WebPSSM$_{X4R5}$**	-	-	-	80.8 (+15.4)	62.3 (-18.9)	0.72 (-0.02; p=0.42)	93.8 (0)	83.2 (-1)	0.89 (-0.01; p=0.47)
**WebPSSM$_{SI/NSI}$**	-	-	-	71.4 (+20.9)	75.5 (-26.4)	0.73 (-0.03; p=0.38)	93.8 (0)	84.1 (-0.9)	0.89 (-0.004; p=0.47)
**WebPSSM$_{SI/NSI}$-C**	79.4 (0)	93.3 (-16.4)	0.86 (-0.08; p=0.10)	-	-	-	-	-	-
**CoRSeq$_{V3-C}$**	100 (0)	76.9 (-0.5)	0.88 (-0.01; p=0.48)	-	-	-	-	-	-
